# Fabrication of a Definitive Obturator for a Patient With a Maxillary Defect: A Case Report

**DOI:** 10.7759/cureus.50578

**Published:** 2023-12-15

**Authors:** Ahmad E Farghal

**Affiliations:** 1 Department of Substitutive Dental Sciences, Taibah University, Medina, SAU

**Keywords:** palatal defect, obturator, prosthodontics, definitive obturator, partial maxillectomy

## Abstract

Maxillectomy defects can lead to oroantral communication, causing difficulties with chewing, swallowing, speech, and facial appearance. Prosthodontists play a crucial role in rehabilitating such defects using obturators. This case report presents the fabrication of a definitive obturator with a cast metal framework for a patient who had an acquired maxillary defect and previously experienced issues with an ill-fitting obturator. In this clinical report, the patient's canine teeth on both sides and the premolars and molars on the left side were used for rest placement. Retention was achieved by utilizing the remaining teeth, employing two embrasure Aker clasps on the left molars and premolars and a C-wrought wire clasp on the right canine. A complete palate was designed as the major connector to ensure optimal load distribution to the surrounding tissues. Additionally, an indirect retainer was planned for the right canine. This definitive prosthesis rehabilitated the patient, improving masticatory efficiency, enhancing speech clarity, and improving quality of life.

## Introduction

There is an increase in the burden of cancer globally due to population growth, aging, and an increase in risk factors such as obesity, smoking, and diet [1]. Head and neck cancers most commonly occur in the oral cavity. Oral cancer's prognosis and survival rates are poor despite significant advancements in its treatment [2,3]. Cancer in the maxillary arch is an uncommon tumor with higher mortality, and 10% of all oral cancers develop in the oral cavity subsites of the upper gingiva and hard palate [4].

Based on the tissue from whence they originated, malignant tumors of the maxilla can be categorized as squamous cell carcinoma, salivary gland tumors such as mucoepidermoid carcinomas, mesenchymal tumors such as chondrosarcomas, and other malignancies, including basal cell carcinoma and malignant schwannoma [5].

Using free flaps and advances in microvascular surgery, many oncology patients with palatal tumors have been able to have their tumors resected and immediately reconstructed after the surgery. A flap with vascularized bone is an ideal option to optimize the future prosthetic bearing area. In the event that the resection site cannot be closed surgically, an obturator must be provided. In addition to improving chewing, swallowing, speech, dental aesthetics, and facial support, the obturator restores the partition between the nasal and oral cavities, thus improving quality of life [6].

Postsurgical maxillary defects can result in several problems, such as hypernasal speech, nasal fluid leakage, the high potential for aspiration, poor aesthetics, and impaired masticatory function [7]. Therefore, treatment of the maxillary defects through surgery or prosthodontics is crucial to these patients' recovery. Some oncology patients may require conventional rehabilitation with an obturator following surgery [8].

Patients who have had a maxillectomy typically undergo several stages of prosthetic treatment. First, a surgical obturator is made and worn for the first one to four weeks after the procedure. Next, an interim obturator is made and worn for three to six months until the defect is improved, and finally, a long-term obturator is made [9].

In the initial postoperative phase, a surgical obturator acts as a partition between the oral and nasal cavities, enabling relatively normal speaking and deglutition and minimizing the psychological effects of the operation and the hospital stay. Additionally, it offers a matrix for surgical packing and lowers the chance of surgical wound contamination [10].

After the surgery, the surgical obturator can be adjusted to accommodate changes in the defect and surrounding tissues. In the meantime, an interim obturator can assist with oral functions until the wound has fully healed and the defect has achieved stability in terms of shape and size. Once the maxillary defect has healed and become stable, a permanent obturator can be used for long-term restoration. An effective seal of the defect is crucial to preventing liquid leakage into the nasal canal. Removable prostheses must be constructed with adequate support, retention, and stability to ensure proper functionality. The type and size of the defect, the presence of supporting palatal shelves, and the condition of the remaining dentition are essential factors that influence the movement of the prosthesis during use. In cases of incomplete dentition, the remaining teeth can serve as abutments, improving the prognosis of the prosthesis [10].

Care must be taken to prevent overload of the remaining dentition and to retain these teeth to the best of their ability. Various types of obturators have been used, such as hollow bulbs, full bulbs, and two-piece obturators. Obturators with a hollow design are often preferred for their light weight [11].

This case report discusses the step-by-step process of creating a cobalt chromium obturator, which is a special type of dental device used to close a gap in the palatal bone of the upper jaw. The report focuses on the clinical stages involved in making a one-part hollow box obturator.

## Case presentation

A 70-year-old male patient was referred to the Department of Prosthodontics, Tabiah University Dental Hospital, Madina, Saudi Arbia, with a chief complaint regarding a previously ill-fitting acrylic maxillary obturator. The Research Ethical Committee of the College of Dentistry, Taibah University, Madinah, Saudi Arabia, approved this study (approval # 14032022). The specific issues reported by the patient were inadequate retention of the old obturator, stability, leakage, and food accumulation underneath the obturator. As a result, the patient desired to replace the obturator with a more suitable alternative. The patient had undergone a right maxillectomy due to the surgical removal of squamous cell carcinoma from the right maxillary sinus. Following the surgery, the patient received postoperative radiotherapy. Approximately six years ago, an obturator was fabricated for the patient to obturate the defect caused by the maxillectomy. 

**Figure 1:**
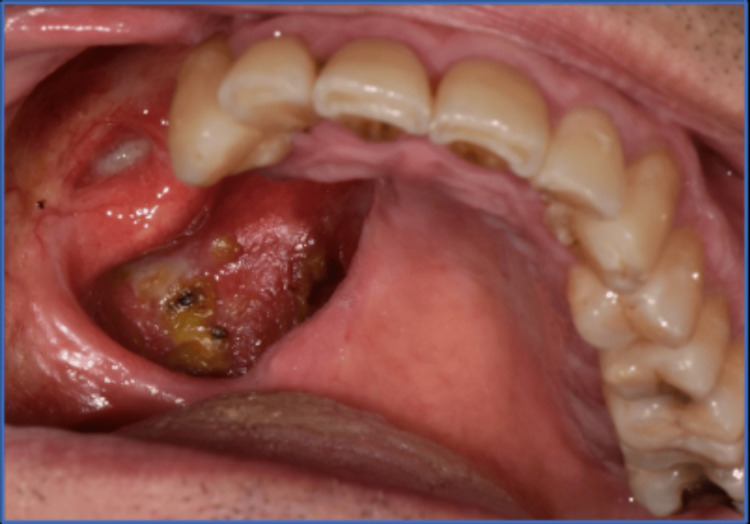
Intra-oral view of the maxillary defect

The extra-oral examination revealed a class III skeletal base, with no abnormalities detected in the examined lymph nodes, temporomandibular joint (TMJ), or face. The intra-oral examination revealed a surgical defect on the right side of the hard palate resulting from a right maxillectomy. According to Aramany's classification of maxillary defects, this defect is classified as Class II [12]. The gingiva on the intact side and lower arch appeared healthy, displaying pink, but with generalized recession. The remaining teeth exhibited a 16% bleeding index and a 34% plaque index. The occlusal examination revealed a Class III malocclusion, characterized by a 0.5 mm anterior open bite and a group function occlusion when the obturator was in place during both centric and eccentric occlusions. Additionally, there was a slight midline shift to the right side (Figure 1).

The patient's diagnosis includes an acquired palatal defect resulting from the surgical removal of a tumor, generalized plaque-induced gingivitis, acquired tooth loss, and a sub-optimal maxillary obturator that is causing leaks. The primary goal of the treatment was to close the communication between the oral and nasal cavities using an obturator. This would artificially block the unrestricted transfer of speech sounds, food, and liquids between these cavities. Additionally, the treatment aimed to enhance the aesthetics and function of the patient's oral cavity.

The proposed course of treatment involved giving the patient oral health instructions (OHI), performing both supra and subgingival scaling and polishing, offering guidance on using floss and interdental brushes, recommending a fluoridated mouthwash with 0.05% sodium fluoride (NaF), and suggesting the use of a toothpaste with a minimum of 1350 parts per million (ppm) of fluoride. Following these interventions, the plan was to provide the patient with a removable cobalt-chrome partial obturator for the maxilla.

**Figure 2:**
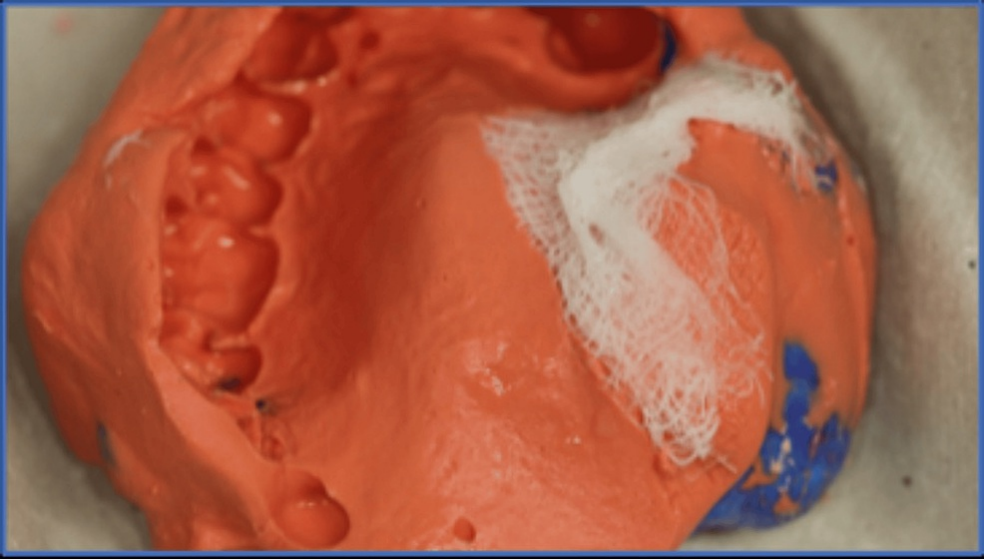
Primary impression of the maxilla

The maxillary and mandibular impressions were taken using irreversible fast-setting hydrocolloids (Tropicalgin, Zhermack) after modifying the upper stock tray to ensure a better fit and to block out undercuts with petrolatum-laden gauze. These impressions were poured with dental stone type IV to produce study casts (Figure 2).

The maxillary cast was duplicated for future reference. The study casts were accurately surveyed to determine the design of the metal framework. Considering his functional and aesthetic requirements, a removable cobalt-chrome partial obturator for the maxillary arch was planned. Following the jaw relation record, the casts were mounted on a semi-adjustable articulator.

**Figure 3:**
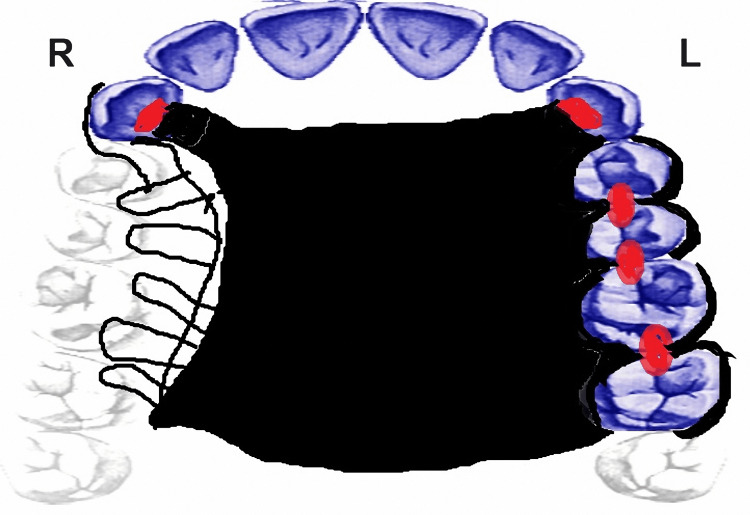
Design drawn for the removable cobalt-chrome partial obturator

The remaining teeth and palate provided the necessary support. Both sides' canines, left-side premolars, and molars were used for cingulum and occlusal rest placement. Retention was achieved by utilizing the remaining teeth, with two embrasure Aker clasps on the left molars and premolars and a C-wrought wire clasp on the right canine. The placement of cingulum rest as an indirect retainer was planned in the right canine tooth. To ensure the functional load was evenly distributed, it was determined that the remaining palate should be fully covered (Table 1 and Figure 3).

**Table 1:** Design for the removable cobalt-chrome partial obturator used in the case presented.

	Right side	Left side
Guide plane	Distal proximal surface of canine	None
Rests	Cingulum rest on canine	Cingulum rests on the canine; mesial and distal occlusal rests on the first premolar, second premolar, and first molar; and mesial occlusal rests on the second molar.
Clasps	wrought wire clasp on canine	Occlusally approaching clasps on the first premolar, second premolar, first molar, and second molar.
Reciprocal components	Part of the major connector on palatal surface of canine	Part of the major connector on the first premolar, second premolar, first molar, and second molar.
Major connector	Palatal plate major connector	

**Figure 4:**
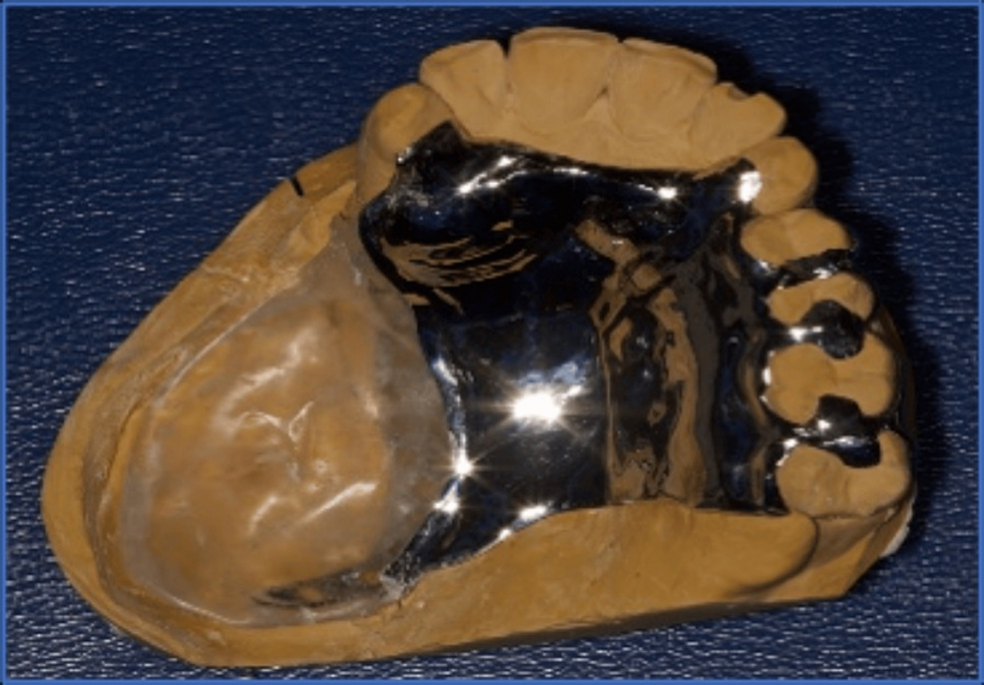
Metal framework of the obturator with all components in their place in the dental cast.

A special tray was made using cold-cure acrylic resin (Acrostone, Egypt) on the primary cast. Border molding was done using green stick compound (Dental Kerr Impression Compound, USA), and the final impression was taken using polyvinyl siloxane (PVS) material (Addition Silicon, Aquasil, Dentsply). The impression was poured with extra-hard type IV dental stone (Kimberlit, Type IV Dental Stone, Protechno-Spain) to generate the master cast. This master cast was duplicated to generate the refractory cast made of investment material, on which the framework wax-up was performed. The framework was then cast using cobalt-chromium alloy (Metal Brealloy, CO-CR alloy, Breadent-Germany) (Figure 4).

**Figure 5:**
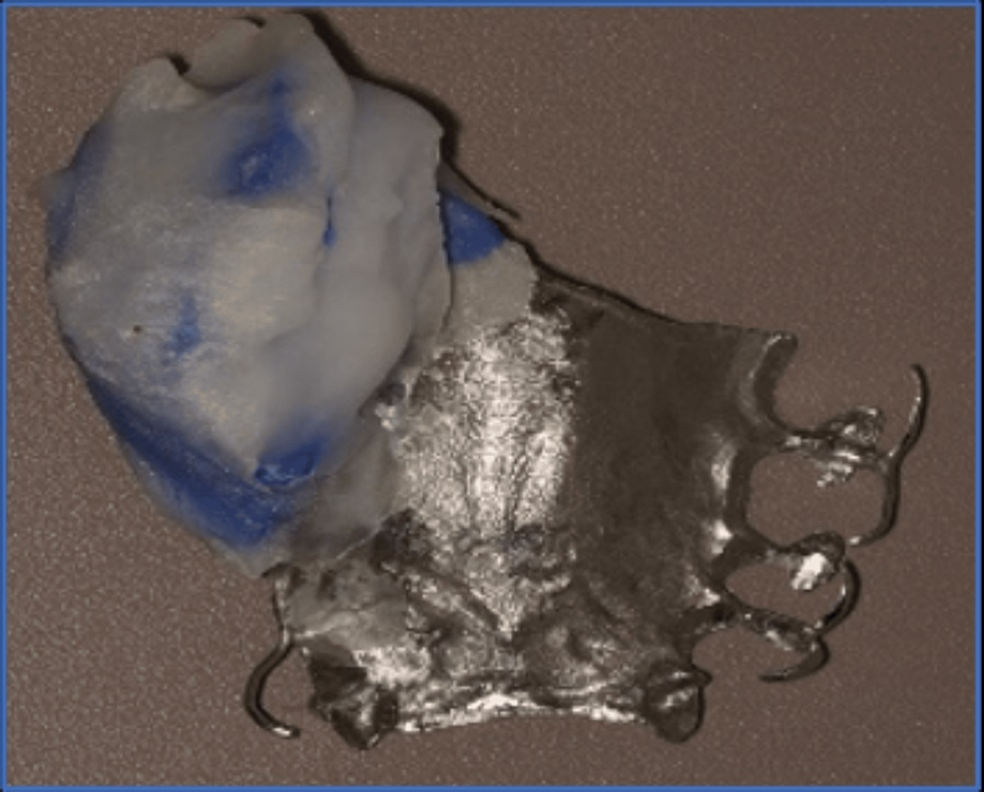
Metal farmwork used the altered cast impression technique for the defect side using PVS impression material.

**Figure 6:**
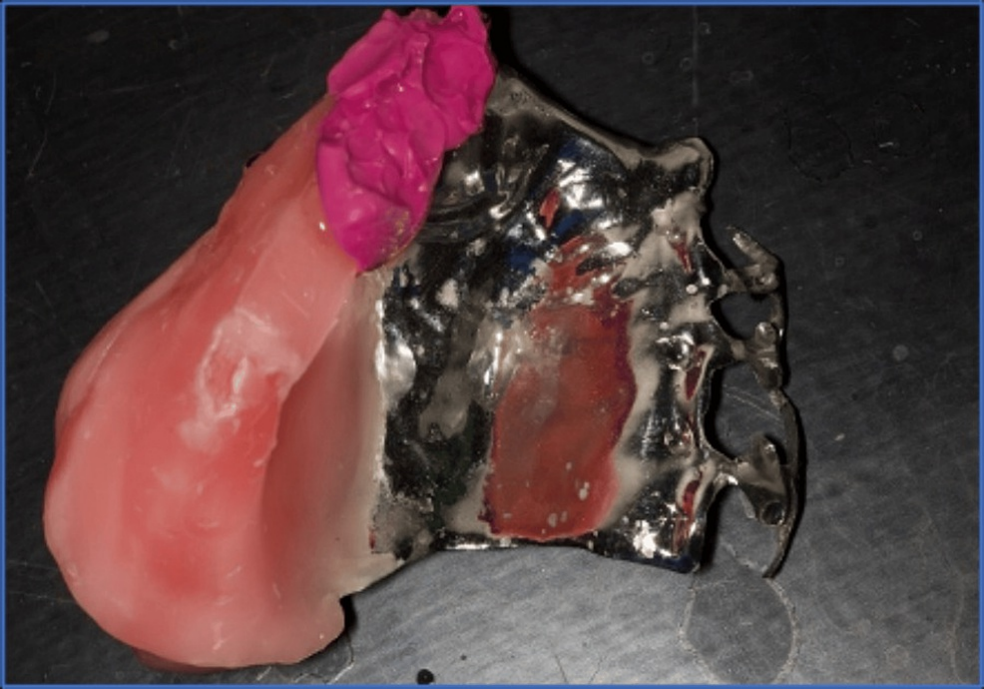
Demonstrates the addition of wax to the metal framework, which is then utilized for recording the jaw relation.

**Figure 7:**
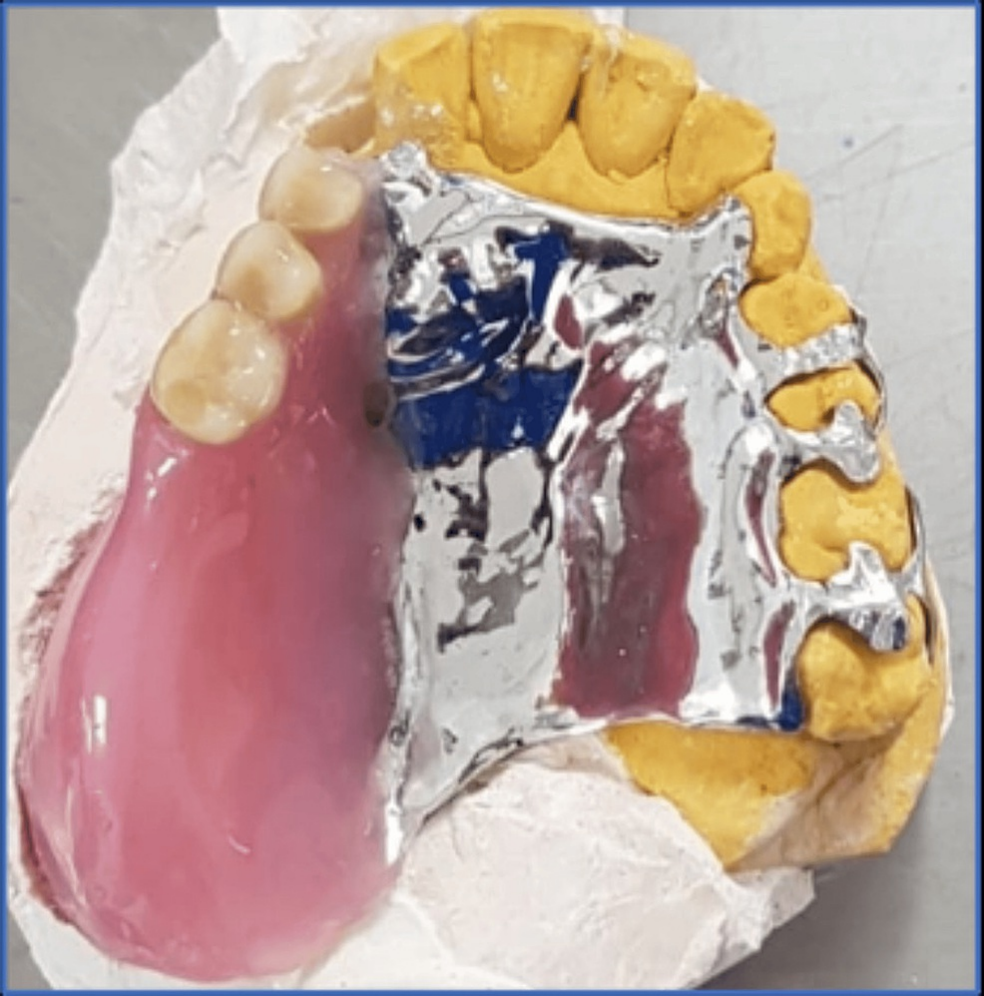
Photograph of the final processed deﬁnitive obturator on the dental cast.

**Figure 8:**
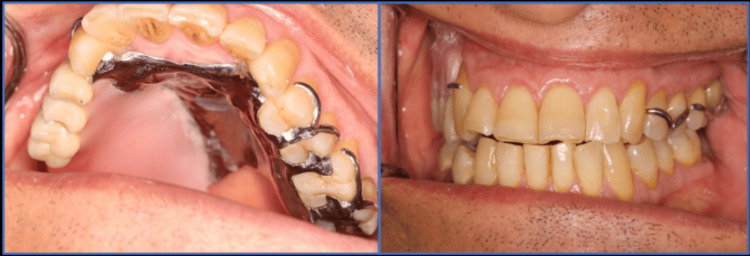
Intraoral views of the final obturator in place

The modified cast technique used PVS impression material to create a precise impression (Figure 5). The fit of the framework with the underlying structures was evaluated by placing it in the patient's mouth and using a pressure indicator paste to assist in the assessment. Occlusion rims were fabricated on the framework, and the centric jaw relation was recorded (Figure 6). The casts were then mounted on a semi-adjustable articulator (Bio-art semi-adjustable articulator. SM66297. Brazil). Acrylic denture teeth (Trubyte, Dentsply, Gloucestershire, England) were arranged, and the obturator was tested to ensure proper occlusion with the mandibular teeth, aesthetic appearance, and support for the underlying tissues. Subsequently, the obturator was processed, finished, and polished following standard procedures (Figure 7). During the insertion, pressure indicator paste (PIP) was used to identify any areas of excessive pressure. The denture was placed in the patient's mouth (Figure 8), and instructions were provided on the care and usage of the obturator. The patient underwent monthly evaluations for the first three months, followed by visits every three months for two years.

## Discussion

Individuals who have undergone a maxillectomy often encounter recurring challenges in prosthodontic treatment related explicitly to insufficient support, retention, and stability. The extent of the defect, the number of remaining teeth, the amount of remaining bone structure, the condition of the surrounding mucosa, the impact of radiation therapy, and the patient's ability to adjust to the prosthetic device all play a role in determining the outlook for prosthodontic treatment in these individuals [13]. Saving as many remaining teeth as feasible for patients undergoing unilateral maxillectomy may be vital for optimal prosthesis design and performance [14]. The other components are subjected to continual pressure from such a massive, hefty obturator, impairing tissue health, patient function, and comfort.

After the obturator has been processed into acrylic resin, the bulb component is frequently hollowed out to reduce the overall weight of the prosthesis. The extent of the maxillary defect determines whether a hollow maxillary obturator is appropriate. By incorporating a hollow design, the weight of the prosthesis can be reduced by as much as 33% [15].

The obturator prosthesis is critical to recovering oral function in postsurgical maxillectomy patients. Framework designs for obturators may differ depending on the defect classification system [16]. Removable obturator prostheses should adhere to fundamental prosthodontic principles, which include distributing stress over a wide area, employing a rigid major connector for cross-arch stabilization, and incorporating stabilizing and retaining components at strategic locations within the arch to minimize the risk of displacement due to functional forces. In this case, a tripodal design was chosen. The remaining teeth, palate, and specifically prepared rests offered support for the prosthesis. Rests were created on the left first and second premolars, the first and second molars, and the right canine on the right side. The complete palate was designed to ensure optimal distribution of functional loads across the underlying tissue [16]. 

In patients with remaining natural teeth, these teeth play a crucial role in maintaining, supporting, and stabilizing the obturator. Retention can be achieved through various means, such as utilizing the remaining teeth or ridge, the lateral aspect of the defect, the undercut in the soft tissue, and the scar tissue. Components for stabilization and indirect retention need to be carefully positioned to prevent movement of the portion of the prosthesis that covers the defect.

Occlusion is the key factor in achieving stability for prostheses. It is crucial to ensure that occlusal forces are evenly distributed in both centric and eccentric jaw positions to minimize prosthesis movement and the resulting forces on individual structures. To reduce stress caused by lateral forces, proper selection of an occlusal scheme, elimination of premature occlusal contacts, and the use of stabilizing components that provide broad distribution are essential [12,17].

A metal framework obturator prosthesis offers several advantages, including its durability and ability to conduct heat, allowing normal stimulation of the supporting structure [18].

It is essential to wait for the defect site's complete healing and dimensional stability before constructing the definitive obturator. The timeframe for this can vary between 3 and 6 months following the surgery, depending on various factors, including the tumor’s prognosis, the defect's size, the progress of healing, and whether teeth are present [16]. The designs of obturators can differ depending on the classification system used to categorize the defects. A tripodal design was chosen in this specific case, considering the support provided by the remaining teeth and palate. The molars, first premolar, and canine were all stabilized to enhance stability. The remaining palate was also covered to ensure proper distribution of functional loads during oral functions.

Dental implants have revolutionized the field of prosthodontics, playing a crucial role in removable [19-21], fixed [22-24], and maxillofacial prosthesis [25,26]. With their ability to provide stability, functionality, and aesthetic appeal, dental implants have transformed the lives of countless individuals, restoring their oral health and overall well-being.

Enhancing the quality of life for hemimaxillectomy patients is a difficult task compared to patients with conventional prostheses. However, specialists with expertise, knowledge, and experience can achieve this goal. By implementing a team approach, utilizing skills and experience at each stage, and regularly evaluating the patient, the challenges faced by hemimaxillectomy patients can be effectively overcome [27].

## Conclusions

The primary challenge in a maxillectomy patient's recovery is ensuring adequate retention, stability, and support. A thorough understanding of the patient's needs and extensive expertise is critical in effectively rehabilitating these individuals. The patient's masticatory abilities, speech intelligibility, and overall quality of life can be significantly improved by designing a definitive obturator prosthesis with maximum coverage and appropriate design.
